# Serotonin 2B Receptor (5-HT_2B_ R) Signals through Prostacyclin and PPAR-ß/δ in Osteoblasts

**DOI:** 10.1371/journal.pone.0075783

**Published:** 2013-09-17

**Authors:** Yasmine Chabbi-Achengli, Jean-Marie Launay, Luc Maroteaux, Marie Christine de Vernejoul, Corinne Collet

**Affiliations:** 1 INSERM UMR606, Hôpital Lariboisière, Paris, France; 2 Université Paris Diderot Sorbonne Paris Cité, Paris France; 3 Service de Biochimie, Hôpital Lariboisière, Paris, France; 4 INSERM U942, Hôpital Lariboisière, Paris, France; 5 INSERM UMR-S839, Institut du Fer à Moulin, Paris, France; Institut de Génomique Fonctionnelle de Lyon, France

## Abstract

Osteoporosis is due to an imbalance between decreased bone formation by osteoblasts and increased resorption by osteoclasts. Deciphering factors controlling bone formation is therefore of utmost importance for the understanding and the treatment of osteoporosis. Our previous *in vivo* results showed that bone formation is reduced in the absence of the serotonin receptor 5-HT_2B_, causing impaired osteoblast proliferation, recruitment, and matrix mineralization. In this study, we investigated the signaling pathways responsible for the osteoblast defect in 5-HT_2B_R^−/−^ mice. Notably, we investigated the phospholipase A2 pathway and synthesis of eicosanoids in 5-HT_2B_R^−/−^ compared to wild type (WT) osteoblasts. Compared to control osteoblasts, the lack of 5-HT_2B_ receptors was only associated with a 10-fold over-production of prostacyclin (PGI_2_). Also, a specific prostacyclin synthase inhibitor (U51605) rescued totally osteoblast aggregation and matrix mineralization in the 5-HT_2B_R^−/−^ osteoblasts without having any effect on WT osteoblasts. Prostacyclin is the endogenous ligand of the nuclear peroxisome proliferator activated receptor ß/δ (PPAR-ß/δ), and its inhibition in 5-HT_2B_R^−/−^ cells rescued totally the alkaline phosphatase and osteopontin mRNA levels, cell-cell adhesion, and matrix mineralization. We conclude that the absence of 5-HT_2B_ receptors leads to the overproduction of prostacyclin, inducing reduced osteoblast differentiation due to PPAR-ß/δ -dependent target regulation and defective cell-cell adhesion and matrix mineralization. This study thus reveals a previously unrecognized cell autonomous osteoblast defect in the absence of 5-HT_2B_R and highlights a new pathway linking 5-HT_2B_ receptors and nuclear PPAR- ß/δ via prostacyclin.

## Introduction

Osteoporosis is a major health problem in developed countries. As the population ages, fracture incidence is set to increase over the coming decades. Bisphosphonates, potent inhibitors of osteoclast-mediated bone resorption, are the conventional treatment for osteoporosis, but present some undesired side effects. In the future, the hope is to propose anabolic treatments for osteoporosis, area in which new targets, based on the serotonergic system, could be an interesting approach.

Serotonin (5-hydroxytryptamine, 5-HT), is a key neurotransmitter that modulates a wide variety of functions in both peripheral organs and the central nervous system (CNS). The different roles of 5-HT involve seven families of 5-HT receptors (5-HTRs): six of them are G protein-coupled, whereas the 5-HT_3_Rs are ionotropic [1]. Although the 5-HT_2B_R is present in the CNS and involved in impulsiveness [2], it has mainly been studied peripherally: the impact of 5-HT_2B_R has been investigated in cardiac [3], pulmonary [4], hepatic [5], erythropoietic [6] and bone [7] systems. These previous studies reported that low-dose serotonin had a proliferative action on chicken periosteal fibroblasts via the 2B receptor [8]. A finding was not consistent with the antiproliferative action of serotonin on osteoblasts via the 5-HT_1B_R reported by *Yadav et al* [9]. We have previously shown that the 5-HT_2B_R is involved in the recruitment of osteoprogenitors, and also in their proliferation and mineralization. In mice, the absence of 5-HT_2B_R induces osteopenia due to impaired bone formation that deteriorates with age [10]. We decided in this study to explore the signaling pathway of this receptor in osteoblasts. Various transduction pathways have already been described for 5-HT_2B_Rs [11]: for instance the stimulation of cell survival via PI3K [12], and of cell proliferation via the Ras-MAPK (mitogen-activated protein kinase) pathway either directly [13], or through transactivation of receptor tyrosine kinase (RTK) [14]. However, the pathway linking 5-HT_2B_R to osteoblast proliferation and differentiation has not yet been precisely defined. During the differentiation of the murine mesoblastic cell line C1 in the osteogenic program, a 5-HT_2B_R-dependent phospholipase A_2_ (PLA_2_) pathway is favored [15]. We therefore hypothesized that this pathway could be important in osteoblast differentiation, and so we investigated the PLA2 pathway with the aim of determining the signaling pathways that leads to reduced osteoblast proliferation and differentiation in murine 5-HT_2B_R^−/−^ primary calvarial cultures. Using both pharmacological and genetic approaches, we observed a hitherto undescribed signaling pathway for the 5-HT_2B_R in the osteoblasts.

## Materials and Methods

### Animals

5-HT_2B_R knockout mice have been already described [14]. The 5-HT_2B_R knockout mice had a 129S2/SvPas (129S2) background. The wildtype (WT) 129S2 background mice used as controls were purchased from Charles River Laboratories (L’Arbresle, France). The wild-type 5-HT_2B_R knockout mice were housed 6 per cage, under a 12/12 h light dark cycle at 21°C, and allowed free access to water and chow from the beginning of the experiment in full compliance with French government animal welfare policy and with European Directive 86/609/EEC. The experiments complied with the Guidelines for Animal Experimentation issued by the local Ethics Committee on Animal Care and Experimentation (Ethical committee Lariboisière-Villemin, Paris, France).

2- to 3-day-old mice were sacrified using guillotine. The calvaria were harvested for cell culture. There is no manipulation prior to sacrifice. YCA/CC have a personal licence from the french veterinary services which allow us animal experimentations.

### Primary calvarial cultures

Primary cultures of mouse osteoblastic cells were obtained by sequential collagenase IV (Sigma–Aldrich) digestions of calvaria from 2- to 3-day-old mice, as previously described [16]. Depending on how they were to be used, the cells were plated at various different cell densities in the osteoblast differentiation medium (*i.e.* α-Minimal essential medium (α-MEM) supplemented with 2 mM L-glutamine (Invitrogen, Cergy-Pontoise, France), 100 IU/mL penicillin/100 mg/mL streptomycin suspension (Invitrogen), 50 µmol/L ascorbic acid and 10 mmol/L, ß-glycerophosphate, Sigma–Aldrich, (Lyon, France). This medium was also supplemented with 8% dialyzed FCS (Invitrogen) with a concentration of serotonin below 2 nM, to study only the 5-HT_2B_R constitutive activity, accounting mainly for the 5-HT_2B_R^−/−^ phenotype [10] and not the other 5-HT receptor activities. The medium was changed every 3 to 4 days. For the mineralizing experiments, cells were plated in 6-well plates at 1×10^6^ cells/plate and grown for 18 days. Cells were then fixed in 4% PFA, and mineralized nodules were stained with Alizarin Red (Sigma–Aldrich) and counted automatically using a software package (Microvision Instruments, Evry, France).

### Alkaline phosphatase-positive colony-forming unit (CFU-F_ALP+_) assays

CFU-F_ALP+_ cells were assayed from the femurs and tibiae of 2- month-old mice as described previously [10]. Briefly, after culturing for 4 days, 100 µg/ml ascorbic acid was added to the culture medium until the end of the experiment. After 11 days, cell colonies were fixed and stained for ALP by adding the Sigma fast substrate buffer bromochloroindoyl-phosphate/nitroblue tetrazolium chloride (Sigma-Aldrich Corp.). The number of CFU-F_ALP+_ cells per dish was counted.

### Treatments

WT cells were treated from the first day to the end of the culture. To determine the role of the 5-HT_2B_ receptor in osteoblast differentiation RS-127445 (20 nM), a specific 5-HT_2B_R antagonist was used. Treatments for phenotype rescues were performed by treating 5-HT_2B_R^−/−^ cultures with an antagonist of PPAR β/δ, GSK0660 (100 nM and 1 µM). The prostacyclin synthase inhibitor U51605 was also used (Cayman Chemicals, Montlucon, France). All the chemicals were purchased from Sigma-Aldrich, except the RS-127445 (Tocris bioscience).

### Cell aggregation assay

Cell-cell adhesion was assessed using a cell aggregation assay [17] with minor modifications. Cells were cultured in bacteriological grade tissue culture wells for 24 hours, and incubated for 60 min at 37°C on a gyratory shaker to allow aggregation. Cell aggregation was evaluated using the N0/N60 index, where N0 is the total number of cells per well and N60 is the total number of particles (*i.e.* single cells and cell clusters) per well after 60 min of incubation.

### Western blotting

Fifteen micrograms of proteins were resolved on 8% acrylamide gel, and then transferred onto polyvinylidene difluoride-Hybond-P membranes (Hybond-P, GE Healthcare). The blots were then probed with primary antibodies for cPLA2 (abcam ab58375) COX_1_ (1:500) (ab695), COX_2_ (1∶500) (ab62331), PPARß/δ (Santa Cruz biotechnology sc:7197), PGIS (1:50) (ab79846), and GAPDH (1:500) (sc:32233). After incubating overnight at 4°C, the membranes were washed and then incubated with horseradish peroxidase-conjugated secondary antibody. The signals were visualized using the West pico ECL system (Thermoscientific, France).

### RT qPCR analysis

For mRNA extraction from cell cultures, we used RNeasy lipid tissue mini kits (Qiagen Courtaboeuf, France). Total RNAs were reverse transcribed into cDNA using the cDNA verso kit (ABgene, Courtaboeuf, France). Quantitative real-time PCR expression analysis was performed on a Lightcycler 480 (Roche Diagnostics, Meylan, France) using Absolute SYBR Green mix (ABgene) at 56°C for 40 cycles. Primers were designed from the online mouse library probes of Roche Diagnostics. Cyclin D1, Runx2, alkaline phosphatase, osteocalcin and osteopontin expressions were measured and normalized, using Aldolase and HPRT as housekeeping genes (**Table 1**).

**Table 1:** 

	Forward	Reverse
**Alkaline phosphatase NM_007431.2**	**TGCTTCATGCAGAGCCTGC**	**TCCTGACCAAAAACCTCAAAGG**
**Osteocalcin NM_007541.2**	**CTCACAGATGCCAAGCCCA**	**CCAAGGTAGCGCCGGAGTCT**
**Runx2 NM_009820.4**	**TTGACCTTTGTCCCAATGC**	**AGGTTGGAGGCACACATAGG**
**Cyclin D1 NM_007631.2**	**TCTTTCCAGAGTCATCAAGTGTG**	**GACTCCAGAAGGGCTTCAATC**
**Osteopontin NM_001204201.1**	**CCCGGTGAAAGTGACTGATT**	**TTCTTCAGAGGACACAGCATTC**
**Aldolase NM_009657.3**	**TGAAGCGCTGCCAGTATGTTA**	**GGTCGCTCAGAGCCTTGTAGA**
**HPRT NM_013556.2**	**GTTGGATATGCCCTTGACTATAATGA**	**CAACATCAACAGGACTCCTCGTATT**

### Quantification of eicosanoids

Eicosanoids were measured in the supernatants of primary WT and 5-HT_2B_R^−/−^ cultures. 6-K-PGF1α (the stable metabolite of prostacyclin), thromboxane B2 (the stable metabolite of thromboxane A2) and leukotriene B4 were measured by ELISA (Cayman chemical, Ann Arbor, MI) the specificity of which was checked using gas chromatography/mass spectrometry [18]. Prostaglandin E2 (PGE_2_) was measured by liquid chromatography-tandem mass spectrometry [19].

### Prostacyclin synthase assay

For the determination of prostacyclin synthase (PGIS) activity, PGH2 (1 µM final concentration) was added to the culture media for 10 min at room temperature. The reaction was stopped by adding NaCl/citric acid 2 M. An acidic ether extraction was subsequently performed. The upper acidic phase, containing the products of the enzymatic reaction, was removed and placed in a clean test tube. Finally, the solution was evaporated to dryness by vacuum centrifugation in order to remove any trace of organic solvent and the pellet was resuspended in the ELISA buffer and the 6KPGF1α content determined.

### cAMP assay

The cAMP level was measured in WT and 5-HT_2B_R−/− cells, as previously described [16]. Briefly the cells were scraped off and then pelleted by centrifuging. Cellular cAMP content was measured by radioimmunoassay and normalized for protein content, which was determined by a bicinchoninic acid assay (Pierce). The IP receptor binding was performed according to a previous report [20].

### Statistical analysis

Statistical analysis was performed using StatView 4.5 software (Abacus Concepts Inc., Berkeley CA, USA). Statistical differences between the experimental groups were assessed by an analysis of variance (ANOVA). The significance threshold was set at p<0.05. All values are shown as mean +/− SEM.

## Results

### Cell-cell adhesion and expression profile of osteoblastic markers of 5-HT_2B_R-/- osteoblasts

**Figure 1:**
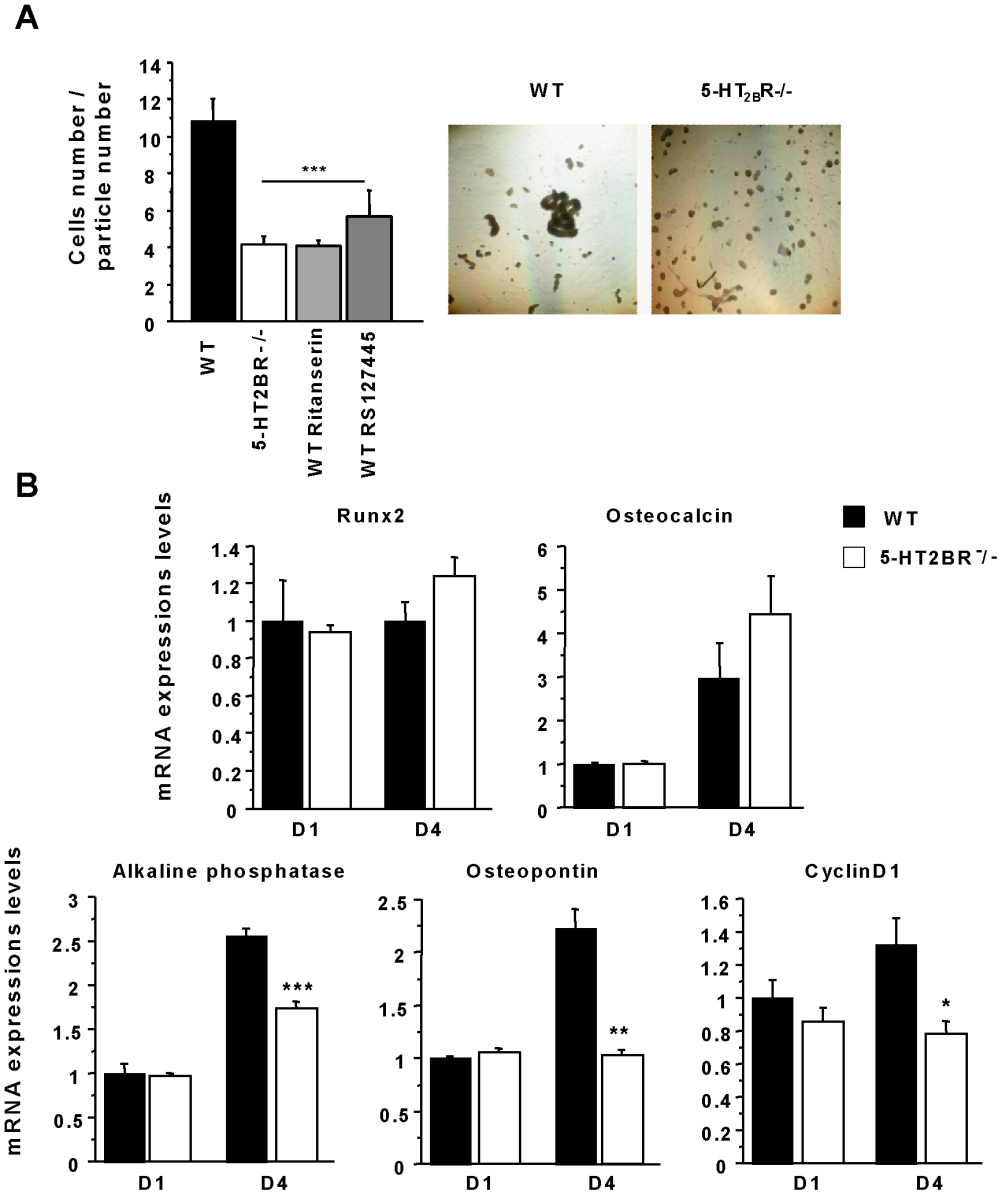
Cell-cell adhesion and expression profile of osteoblastic markers of 5-HT_2B_R-/- osteoblasts. A: Representative pictures of aggregation assays are shown at Day 1 (D1) after the induction of 5-HT_2B_R^−/−^ and WT primary cultures. Counts of cell aggregates were assessed in WT, and 5-HT_2B_R^−/−^ cells, and in WT cells treated with either ritanserin (an inverse agonist of 5-HT_2_Rs) or RS127445 (a selective 5-HT_2B_R antagonist). The 5-HT_2B_R^−/−^ and WT-treated cells displayed a much lower level of cell adhesion than the untreated WT cells. B: the main markers of osteoblast differentiation were measured by RTqPCR. Runx2, osteocalcin expression levels were not modified by the absence of 5-HT_2B_R, whereas cyclin D1, alkaline phosphatase and osteopontin expression levels were decreased. Data are presented as means ± SEM of three independent experiments performed in triplicate. ** p<0.005 *vs* WT, * p<0.05 *vs* WT.

We previously reported [10] that both proliferation assessed by thymidine incorporation and mineralization, assessed by Ca2+/protein ratio, were reduced in 5-HT_2B_R^−/−^ primary calvaria cultures. Since morphologic obsfffervation of self-assembling behavior seemed to be lower in 5-HT_2B_R^−/−^ osteoblasts, we decided to analyze the cell-cell adhesion of 5-HT_2B_R^−/−^ osteoblasts. Cell aggregation experiments were performed after culturing WT and 5-HT_2B_R^−/−^ calvarial osteoblast progenitors for 24 hours. The 5-HT_2B_R^−/−^ aggregates (N0/N60  =  4.12±1.5) contained fewer cells than the WT aggregates (N0/N60  =  10.86±3.8 p<0.001) (Fig. 1A). Similar experiments were performed in WT cultures treated with ritanserin, a 5-HT_2_R inverse agonist, and RS127445, a selective 5-HT_2B_R antagonist. Both ritanserin (100 nM) and RS127445 (20 nM) diminished cell aggregation to a degree similar to that observed in 5-HT_2B_R^−/−^ calvarial cultures (Fig. 1A).

In order to further characterize the phenotype of 5-HT_2B_R^−/−^osteoblasts, we investigated the mRNA level of the main markers of osteoblastic proliferation and differentiation one [D1] and four [D4] days after inducing osteoblastic differentiation. The lower 5-HT_2B_R^−/−^ osteoblast proliferation was confirmed by the reduced cyclin D1 expression at both times (**Fig.** **1B**). With regard to the expression of osteoblastic differentiation markers, such as Runx2 or osteocalcin, we found no difference; while the alkaline phosphatase and osteopontin levels were lower at D4 (**Fig. 1B**). This demonstrates that 5-HT_2B_Rs are involved in the induction of cell-cell osteoblastic adhesion, and are necessary for osteoblast proliferation and mineralization.

### Prostacyclin over-production in 5-HT_2B_R^−/−^ osteoblasts

**Figure 2:**
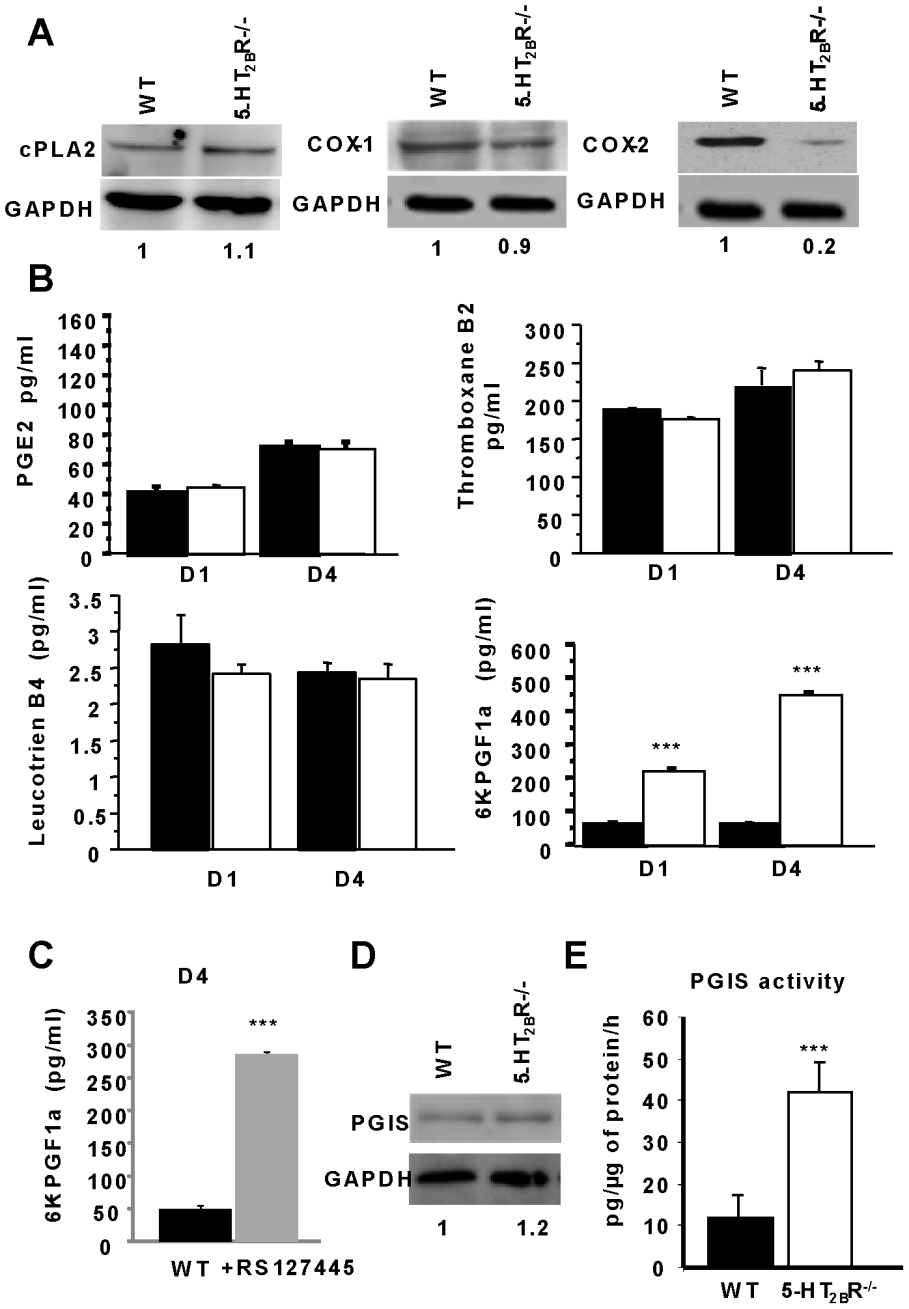
Prostacyclin over-produced in 5-HT_2B_R^−/−^ osteoblasts. A: To determine the role of the PLA_2_ pathway in the 5-HT_2B_R^−/−^ osteoblasts, the expression of PLA_2_ and of COX_1_ and COX_2_ were analyzed by Western blotting. The expression of COX_2_ was markedly lower in the 5-HT_2B_R^−/−^ cells than in the WT cells, whereas no difference was observed in the expression of PLA_2_ or COX_1_. B: Measurement of the main eicosanoids in both genotypes: Prostaglandin E2, Thromboxane B2, Leukotriene B4 and 6-keto prostaglandin F1α, a stable metabolite of prostacyclin, at D1 and D4. Prostacyclin production was greater in 5-HT_2B_R^−/−^ primary cultures than in WT cultures. C: It was also greater in WT cultures treated with RS 127445 at D4. D: Prostacyclin synthase expression was evaluated by Western blotting. Levels of prostacyclin synthase were slightly higher in 5-HT_2B_R^−/−^ than in WT cultures. E: Prostacyclin synthase activity was markedly higher in 5-HT_2B_R^−/−^ than WT cultures. Data are presented as means ± SEM of three experiments performed in triplicate. *** p<0.001 vs. WT.

5-HT_2B_R-dependent regulation of the PLA_2_ signaling pathway has been previously observed in the C1 osteoprogenitor cell line [15]. Using Western blotting to analyze the expression of PLA_2,_ COX_1_ and COX_2_, we observed that PLA_2_ and COX_1_ expressions were unchanged **(** **Fig. 2A** **),** whereas the COX_2_ protein expression level was markedly lower (5-fold) in 5-HT_2B_R^−/−^ cells than in WT cells (**Fig. 2A**). To analyze this pathway more precisely, the different eicosanoid productions were measured, including prostaglandin E_2_ (PGE_2_), thromboxane A2 (TBXA_2_), leukotriene B4 (LTB_4_) and prostacyclin (PGI_2_). We observed no differences according to genotype (**Fig. 2B**), except in the case of 6-keto prostaglandin F1α (6K-PGF1α), the stable metabolite of prostacyclin (PGI_2_). In 5-HT_2B_R^−/−^ primary calvarial osteoblast cultures, the 6K-PGF1α level was considerably higher (4 fold at D1, and 10 fold at D4) than in the WT supernatant (**Fig. 2B**). At D4, WT cells treated with the 5-HT_2B_R antagonist, RS127445 (20 nM), displayed a marked increase (6 fold) in the level of 6K-PGF1α (**Fig. 2C**). We also noticed a significant increase in the expression of the prostacyclin synthase (PGIS) protein, and a four -fold increase in PGIS activity (**Fig. 2D**). Furthermore, the levels of 6 ketoF1α (pg/mL) determined in the serum were significantly higher in 5-HT_2B_R^−/−^ mice than in WT mice: (156.1 +/**−** 24.9 in WT mice *vs.* 261.4 +/**−** 28.7 in 5-HT_2B_R^−/−^ mice; n = 10 in both genotypes p<0.001).

### 5-HT_2B_R−/− osteoblasts phenotype is dependent on prostacyclin overexpression

**Figure 3:**
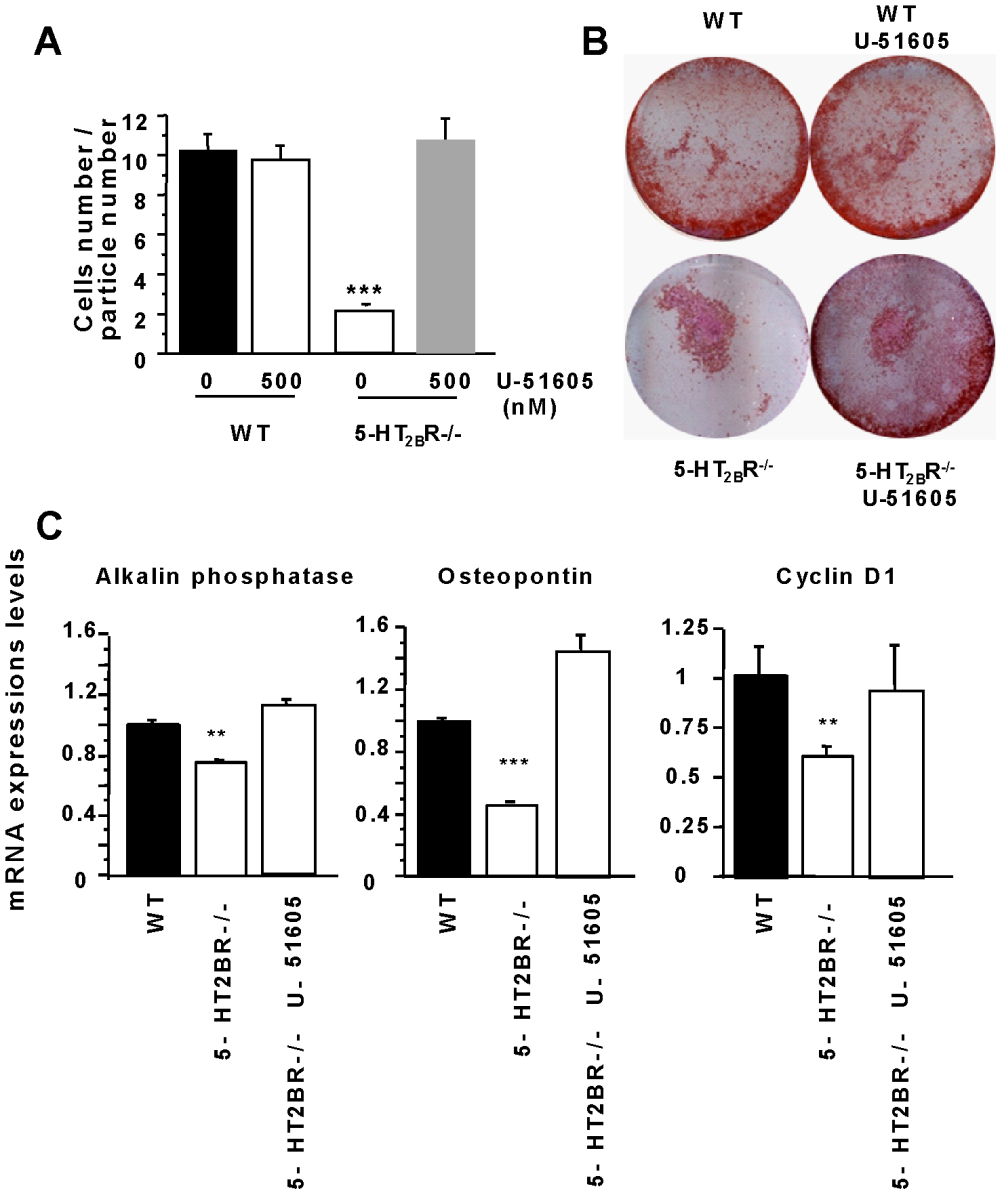
5-HT_2B_R−/− osteoblasts phenotype is dependent on prostacyclin overexpression. A specific inhibitor of prostacyclin synthase (U51605) was used to evaluate the role of the prostacyclin overproduction. A: a cell aggregation assay was performed with this inhibitor on WT and 5-HT_2B_R^−/−^ cells. U51605 had no effect on WT aggregation and restored 5-HT_2B_R^−/−^ cell aggregation. B: mineralization assay: U51605 had no effect on WT cells, but did rescue 5-HT_2B_R^−/−^ mineralization. C: The expressions of the alkaline phosphatase, osteopontin and cyclin D1 level markers modified by the absence of 5-HT_2B_R were restored by adding U51605 to 5-HT_2B_R−/− osteoblasts. Data are presented as means ± SEM of three independent experiments performed in triplicate. ** p<0.05 vs. WT, *** p<0.001 vs. WT.^ $$$^ p<0.005 vs 5-HT_2B_R−/−.

We used a specific prostacyclin synthase inhibitor (U51605) to demonstrate the role of prostacyclin overproduction in the *in vitro* osteoblast phenotype. First, the use of U51605 rescued the cell-cell adhesion (**Fig. 3A** **)** and the differentiation of the 5-HT_2B_R−/− osteoblast cultures **(** **Fig. 3B**). We had also noticed that the U51605 had no effect on the WT osteoblast phenotype (**Fig. 3A and 3B**). We therefore measured the expression of the alkaline phosphatase, osteopontin and cyclin D1 osteoblast markers in the absence or in presence of U51605 in 5–HT_2B_R−/− and WT cultures. The levels of alkaline phosphatase, osteopontin and cyclin D1 mRNA were restored by treating in calvaria cultures with the prostacyclin synthase inhibitor (**Fig. 3C**).

To confirm the link between the prostacyclin overexpression and the bone phenotype previously described in 5-HT_2B_R^−/−^ mice, similar experiments were performed using CFU-osteoblasts. Our first results were confirmed: U51605 had no effect on the WT number of CFU-osteoblasts (WT: 132 +/– 10 *vs* WT with U51605: 128 +/–5 per dish), however this inhibitor did induce a rescue of the 5-HT_2B_R^−/−^ number of CFU osteoblasts (5-HT_2B_R^−/−^ : 48 +/– 4 *vs* 5-HT_2B_R^−/−^ with U51605: 134 +/– 15 per dish).

In summary, these results demonstrate that the absence of 5-HT_2B_R induces prostacyclin overproduction, and its inhibition restores the phenotype of 5-HT_2B_R^−/−^ osteoblasts.

### Pharmacological inhibition of PPAR-ß/δ restores the phenotype of 5-HT2BR-/- osteoblasts

**Figure 4:**
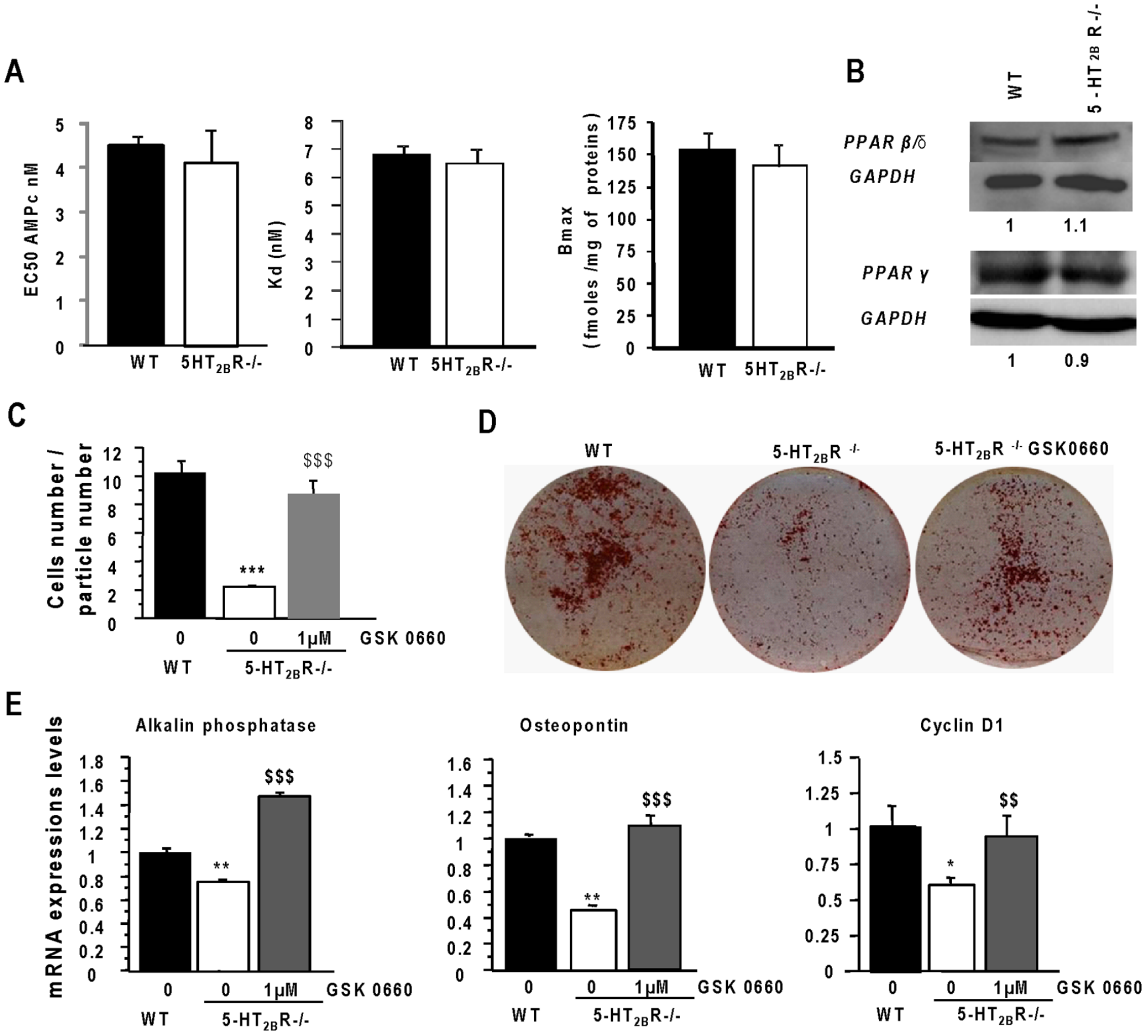
Pharmacological inhibition of PPAR-ß/δ restores the phenotype of 5-HT2BR−/− osteoblasts. PGI_2_ is an endogenous ligand of nuclear PPAR ß/δ, and also can act via the membrane IP receptor. We evaluated the involvement of these two potential prostacyclin targets in 5-HT_2B_R^−/−^ cells. A: For the membrane IP receptor: the cAMP level and the binding parameters of the IP receptors were measured in both cell types. None of these parameters was modified by the absence of the 5-HT_2B_ receptor. B: PPAR-ß/δ and PPAR-γ expressions were analyzed by Western blotting, and found to be unchanged in both cell types. The involvement of PPAR ß/δ was determined using specific antagonist of this receptor. C: An aggregation assay was carried out in the presence of GSK0660 (a specific antagonist of PPAR-ß/δ) for 5-HT_2B_R^−/−^ cultures. The GSK0660 treatment restored cell aggregation in 5-HT_2B_R^−/−^ cultures. D: A mineralization assay (red alizarin staining) was performed under the various different conditions: GSK0660 treatment rescued the phenotype in 5-HT_2B_R^−/−^ osteoblasts. E: Similar results were obtained for the transcriptional expressions of the alkaline phosphatase, osteopontin, and cyclin D1 level markers. Data are presented as means ± SEM of three independent experiments performed in triplicate. *** p<0.005 vs. WT and ^$$$^ p<0.005 vs 5-HT_2B_R−/−.

Prostacyclin is an endogenous ligand of the nuclear peroxisome proliferator-activated receptor (PPAR) ß/δ, and can also act via the membrane IP receptor [21]. We measured the IP receptor-dependent cAMP production, in calvarial primary osteoblasts of each genotype; the binding affinity of the IP receptor (Kd and Bmax) was also evaluated. No difference was observed between the two genotypes for either binding or second messenger production (**Fig. 4A**). Since there is considerable evidence indicating that PPARs play an important role in bone metabolism [22], we analyzed the role of PPAR-ß/δ during the early stages of 5-HT_2B_R^−/−^ cultures. The expression of PPAR-ß/δ was not different in primary 5-HT_2B_R^−/−^ osteoblasts and WT osteoblasts. Moreover, PPAR-γ expression was unchanged between the two genotypes. (**Fig. 4B**).

In order to demonstrate the interaction between the PPAR-ß/δ and 5-HT_2B_R pathways, we attempted to rescue the 5-HT_2B_R^−/−^ phenotype with GSK0660 (1 µM), a specific PPAR-ß/δ antagonist. After GSK0660 treatment of 5-HT_2B_R^−/−^ cultures, the cell aggregation (**Fig. 4C** **)** and differentiation (**Fig. 4D**) were restored. Furthermore, after the GSK0660 antagonist treatment of 5-HT_2B_R^−/−^ cultures, cyclin D1, alkaline phosphatase and osteopontin mRNA expressions were restored to the WT level (**Fig. 4E**).

Finally, we confirmed these results using bone marrow cultures. GSK0660 treatment of 5-HT_2B_R^−/−^ osteoblasts restored the CFU osteoblast number (5-HT_2B_R^−/−^: 48 +/− 4 *vs* 5-HT_2B_R^−/−^ GSK0660: 141 +/− 6 per dish). These findings demonstrated that inhibiting PPAR ß/δ restored the osteoblast phenotype of 5-HT_2B_R^−/−^ osteoblasts.

## Discussion

Our report shows that the osteoblast cell-cell adhesion is impaired in the absence of 5-HT_2B_Rs. Our results validate our hypothesis that the decreased bone formation observed in 5-HT_2B_R^−/−^ mice is cell autonomous. This osteoblast defect of 5-HT_2B_R^−/−^ is associated with prostacyclin overproduction, the inhibition of which restored, a normal osteoblast phenotype. Furthermore, pharmacological inhibition of PPAR-ß/δ in 5-HT_2B_R^−/−^ mice improved osteoblast recruitment and osteoblast marker expression, and reversed the decreased mineralization. This is the first report establishing an indirect link, through prostacyclin overproduction, between 5-HT_2B_R, a seven trans-membrane G-protein-coupled receptor, and PPAR-ß/δ, a nuclear receptor. We show here that at early time points in a calvarial culture, 5-HT_2B_R signaling controls cell-cell adhesion and osteoblast mineralization via the PLA2/prostacyclin pathway. Indeed, the recruitment of 5-HT_2B_R^−/−^ osteoblast precursors from the bone marrow and from primary calvaria cells was lower than in WT cells, with reduced alkaline phosphatase, cyclin D1 and osteopontin levels. Interestingly, the reduced level of osteopontin, which is a sibling protein expressed at a different stage of the osteoblast differentiation pathway, was associated with decreased mineralization.

Besides, we have previously reported that PLA_2_ and NO pathways are activated by 5-HT_2B_Rs during the differentiation of an osteoblast precursor cell line [15]. In the present study, since the PLA_2_ pathway is major during osteoblast differentiation, and PLA_2_ protein expression level was unchanged, we investigated all the effectors downstream PLA_2_. We first showed that the inducible COX_2_ expression was down-regulated in 5-HT_2B_R^−/−^ osteoblast cultures. These results are consistent with the *in-vivo* phenotype of COX_2_ knock-out mice, which present defective bone formation with reduced osteoblastogenesis [23], similar to that observed in 5-HT_2B_R^−/−^ mice [10]. Investigating COX metabolites in 5-HT_2B_R^−/−^ osteoblasts, we observed that PGE_2_, LTB_4_, and TBX_2_ levels remained unchanged at the beginning of the differentiation. Unexpectedly, we also observed over-production of PGI_2_ by 5-HT_2B_R^−/−^ osteoblasts associated with a markedly increased PGIS activity. It is known that the tyrosine nitration of PGIS can inhibit its activity [24]. We have previously shown that in the absence of the 5-HT_2B_R, the production of nitric oxide is reduced [15] [25], and we hypothesized that this could induce an increase of PGIS activity. The exact role of PGI_2_ in osteoblasts remains poorly understood [26] [27], but a recent publication has reported that aged prostaglandin I_2_ synthase knockout [PGIS^−/−^] mice exhibit increased bone formation [28], i.e. the reverse of the bone phenotype observed in our 5-HT_2B_R^−/−^ mice. We showed that the osteoblast phenotype of 5-HT_2B_R^−/−^ mice could be completely reversed *in vitro* by a specific PGI2 inhibitor. Finally, our data suggest that the overproduction of PGI_2_ decreases osteoblast recruitment, proliferation, and differentiation. However, the absence of prostacyclin production has no effect on osteoblast differentiation, suggesting that it may only play a minor role at the physiological level during osteoblast differentiation, but that it can be harmful at high levels.

PGI_2_ is an endogenous ligand of PPAR-ß/δ, and it plays important roles in various metabolic functions, and differs from the two other PPAR isotypes (α and γ) by being more widely expressed. The action of PPAR-ß/δ in bone was poorly investigated, although it has been reported to be expressed in both osteoblasts and osteoclasts [29]–[31], and mice lacking PPAR ß/δ display a decreased bone volume with increased osteoclastic resorption [31].

On the other hand we found that PPAR ß/δ pharmacological inhibitors restore the osteoblastic phenotype of 5-HT_2B_R ^−/−^ mice suggesting that PPARß/δ over activation can also be deleterious for bone metabolism.

Moreover, prostacyclin overproduction is known to trigger PPAR-ß/δ activation and we observed down-regulated osteoblast markers, such as alkaline phosphatase and osteopontin. Furthermore a PPAR-ß/δ antagonist restored the phenotype of 5-HT_2B_R^−/−^ osteoblasts, including Cyclin D1 transcription level. The role of PPARß/δ in proliferation remains uncertain as some studies indicate that PPARß/δ promotes tumorigenesis while others suggest that PPARß/δ attenuates tumorigenesis [32]. It is known that the 5-HT_2B_R enhances cell proliferation via different pathways including cyclin D1 [14], the transcription of which is restored by a PPAR-ß/δ antagonist. Consequently our results showed and confirm that cyclin D1 is an indirect 5-HT_2B_R target maybe via inhibition of PPARß/δ [32].

Reduced recruitment and cell-cell adhesion by PPAR-ß/δ activation have already been described in leukocytes [33]. In fact, PPAR-ß/δ could play a so-far unknown role in osteoblast function via the regulation of numerous genes. Among its targets, we showed that osteopontin was down-regulated by the pharmacological activation of PPAR-ß/δ. Our results indicate for the first time that by inactivating prostacyclin production, the absence of 5-HT_2B_Rs prevents the different steps necessary for osteoblast differentiation to occur.

Our findings reveal a coupling between PPAR-ß/δ and 5-HT_2B_Rs in bone that might well also occur in other tissues, since the plasma level of PGI2 was also increased in 5-HT_2B_R−/− mice. In the heart, 5-HT_2B_Rs regulate cardiac development and function [34]–[35], and PPAR-ß/δ is an essential transcription factor in the myocardial metabolism [36]–[37]. Moreover, prostacyclin treatment improves pulmonary artery hypertension (PAH) patients, suggesting that the 5-HT_2B_R-prostacyclin/PPAR-ß/δ coupling could also be involved in PAH [38]. 5-HT_2B_R activation appears to play a major role in this disorder, and we showed that 5-HT_2B_R^−/−^ mice do not develop PAH after hypoxia [39]. This phenotype might be related to PGI_2,_ since we detected a marked overproduction of 6-keto PGF1a, the stable catabolite of prostacyclin, in the plasma of 5-HT_2B_R^−/−^ mice.

We mainly investigated in our *in vitro* experiments the constitutive activity of 5-HT_2B_R since the effect of the ritanserin, inverse agonist of 5-HT_2_R led to a similar phenotype of 5-HT_2B_R^−/−^ osteoblasts. As the effects of RS127445 and ritanserin in WT osteoblast were similar, these results could therefore be related to a 5-HT_2B_R constitutive activity and its particular pharmacological characteristics are suggested in the publication in Locker *et al* [15]. Indeed, the specific 5-HT_2B_R antagonist RS-127445 behaves as an inverse agonist in our mice model. Since species-species differences in the pharmacological properties of 5-HT_2B_R have been reported [40] and since Locker *et al* suggests that the osteoblast 5-HT_2B_R presents particular pharmacological characteristics in osteoblastic cell lineages [15], Therefore new drugs targeted to 5-HT_2B_R could be developed in order to treat osteoporosis.

In conclusion, our study reveals a markedly impaired osteoblast phenotype in the absence of the serotonin 2B receptor and describes a hitherto-unknown interaction between 5-HT_2B_Rs and the nuclear PPAR-ß/δ receptors via prostacyclin that may have various pathological and physiological implications.
